# TGF-β production by eosinophils drives the expansion of peripherally induced neuropilin^−^ RORγt^+^ regulatory T-cells during bacterial and allergen challenge

**DOI:** 10.1038/s41385-022-00484-0

**Published:** 2022-02-15

**Authors:** Angela Fallegger, Martina Priola, Mariela Artola-Borán, Nicolás Gonzalo Núñez, Sebastian Wild, Alessandra Gurtner, Burkhard Becher, Shida Yousefi, Hans-Uwe Simon, Isabelle C. Arnold, Anne Müller

**Affiliations:** 1 Institute of Molecular Cancer Research, University of Zurich, Zurich, Switzerland; 2 Institute of Experimental Immunology, University of Zurich, Zurich, Switzerland; 3 Institute of Pharmacology, University of Bern, Bern, Switzerland; 4 Department of Clinical Immunology and Allergology, Sechenov University, Moscow, Russia; 5 Laboratory of Molecular Immunology, Institute of Fundamental Medicine and Biology, Kazan Federal University, Kazan, Russia; 6 Institute of Biochemistry, Brandenburg Medical School, Neuruppin, Germany

## Abstract

Eosinophils are best known for their effector functions in settings of parasitic infection or allergen challenge, but have also increasingly been implicated in immune regulation at mucosal sites. Here, we show using bacterial infection and antigen challenge models that extrathymic Foxp3^+^ regulatory T-cells that arise de novo in the context of bacterial infection require an intact eosinophil compartment. Mouse strains with a constitutive or conditional eosinophil deficiency, or with an eosinophil-specific ablation of *Tgfb*, lack bacterially induced neuropilin-negative, RORγt-positive gastrointestinal Treg populations in models of *Helicobacter pylori*, *Helicobacter hepaticus* and *Citrobacter rodentium* infection, as well as in the steady state colon and upon oral ovalbumin challenge. Treg priming in lymph nodes appears not to be impaired. Eosinophil-dependent tissue-resident Tregs express CTLA4, ICOS, CD39 and T-bet in addition to RORγt. Eosinophils reside in close proximity to Tregs in infected tissues, and specifically induce the expansion of newly formed Tregs, but not conventional T-cells in vivo and in vitro. TGF-β expression in eosinophils is induced by bacterial contact and during allergen exposure. Specific *Tgfb* ablation in eosinophils and the associated Treg defects result in excessive T-cell responses in the examined Th2- but not Th1-polarized settings.

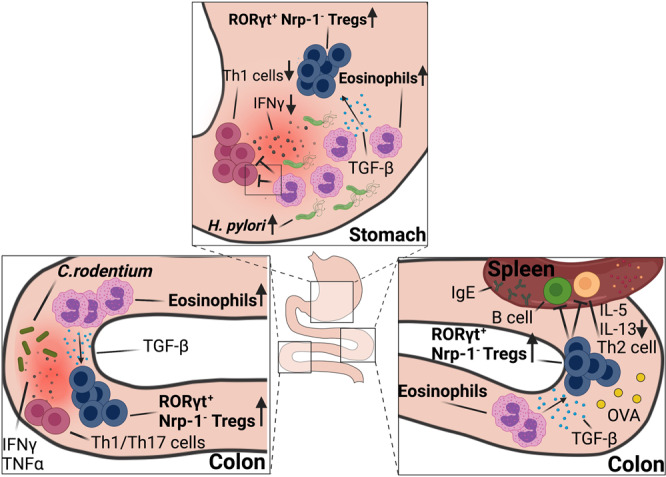

## Introduction

Eosinophils develop and differentiate in the bone marrow, travel via the circulation and reside in a variety of tissues at steady state that include the lung, spleen, uterus, mammary gland, and the lamina propria of the gastrointestinal (GI) tract^1,2^. Eosinophils have both effector/orchestrator functions, which are best understood in settings of allergic reactions and parasitic and bacterial infections, and regulatory activities that appear to be highly tissue- and context-specific^3^. Eosinophils regulate -both positively and negatively- innate and adaptive immune responses through direct physical interactions with other leukocytes and non-immune cells and the activities of their secreted immunomodulatory products^1,3^. Eosinophils regulate various aspects of lymphocyte biology and are themselves subject to lymphocytic regulation. In allergic airway inflammation for example, IL-5 and IL-13 produced by antigen-experienced Th2 cells or type 2 innate lymphoid cells (ILC2s) promote eosinophil production, priming, and survival, and eosinophil recruitment via eotaxin gradients, respectively^4,5^. Eosinophils in turn elicit the expression of the Th2 chemokines CCL17 and CCL22, and the recruitment of effector T-cells to the allergen-challenged lung^6^. We have recently shown that tumor-infiltrating eosinophils produce these and other T-cell chemokines to recruit effector T-cells to the tumor microenvironment and to locally activate anti-tumor immunity^7^. Eosinophils are capable not only of recruiting T-cells, but also of locally activating their clonal expansion in an antigen-dependent manner^8,9^. In certain Th1- or Th17-polarized settings, however, eosinophils may also dampen T-cell responses to maintain tissue homeostasis and to prevent immunopathology associated with bacterial infection^10^; this type of regulatory activity of the eosinophil appears to be most prominent in the GI tract, the numerically most important reservoir of eosinophils in the body. Other functions that have been specifically assigned to GI eosinophils are the generation of IgA-producing plasma cells, stimulation of intestinal mucus secretion, and induction of Peyer’s patch development^11,12^. GI eosinophils remain poorly understood relative to other tissue-resident eosinophil populations^3^ and the mechanisms underlying their GI-specific homeostatic function are largely enigmatic.

Here, we use bacterial infection and oral allergen challenge models to examine a role for eosinophils in shaping the GI regulatory T-cell (Treg) compartment. We find that the constitutive or conditional ablation of eosinophils leads to excessive effector T-cell responses to, and improved control of bacterial infection, while at the same time compromising the parallel development of a bacterially driven Treg response. This function of eosinophils is particularly evident in the gastric mucosa, which at steady state is devoid of Tregs. The eosinophil-specific ablation of TGF-β production phenocopies the effects of loss of the entire lineage, and eosinophil overproduction due to excessive transgenic IL-5 expression has the opposite effect. The Treg-promoting function of eosinophil-derived TGF-β was confirmed in ovalbumin-specific models of de novo Treg generation, and appears to exclusively shape the peripherally induced GI Treg pool, with nor or minor effects on the thymus-derived Treg compartment.

## Results and discussion

### The selective ablation of eosinophils compromises the accumulation of peripherally induced Tregs at sites of bacterial infection

**Fig. 1:**
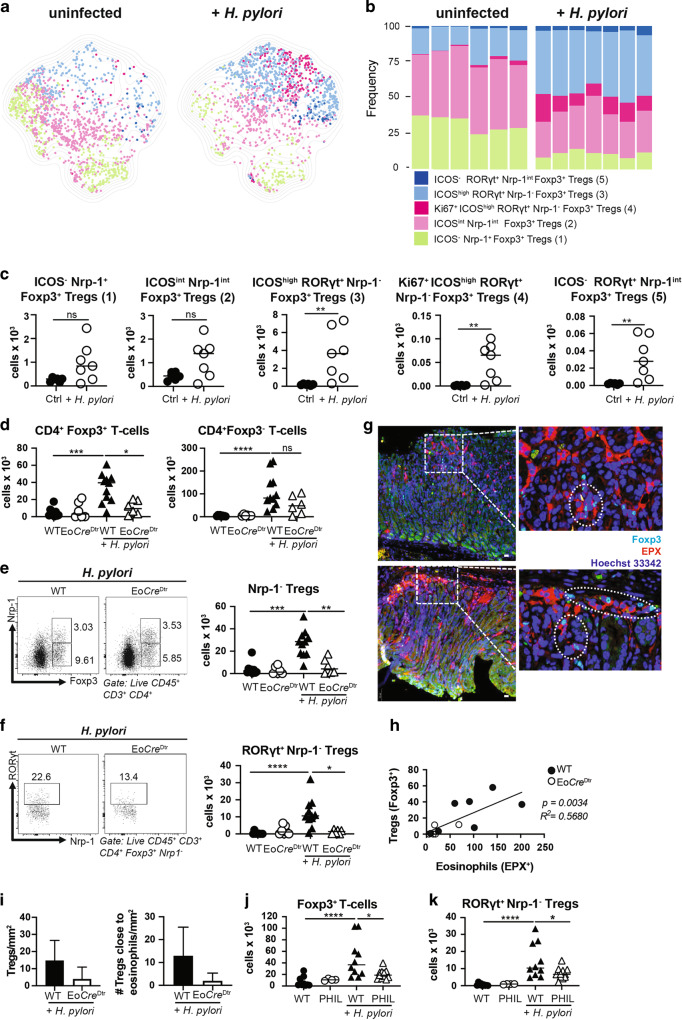
The selective ablation of eosinophils results in reduced Treg populations in infected tissues. **a**–**c** Multidimensional analysis of gastric Treg populations in *H. pylori*-infected and control mice. **a** UMAP of 1000 stochastically selected gastric CD4^+^ Foxp3^+^ Tregs from seven infected (right) and six uninfected mice (left). **b** Frequencies of the five indicated Treg populations of the mice shown in **a**; the color code in **a** and **b** corresponds to the five gastric Treg populations described in the text. **c** Absolute counts of the five Treg populations shown in **a**, **b**. **d**–**i** Eo-*Cre*^DTR^ mice and their wild-type (Cre^-^WT) littermates were infected with *H. pylori* strain PMSS1 for 6 weeks; all mice (WT and Eo-*Cre*^DTR^) received diphtheria toxin (DT) twice a week for the entire time course to deplete eosinophils. Absolute counts of gastric Foxp3^+^ and Foxp3^−^ CD4^+^ T-cells are shown in **d**; absolute counts of gastric Foxp3^+^ Nrp-1^−^ Tregs are shown in **e** alongside representative FACS plots of the two infected groups. Absolute counts of gastric RORγt^+^ Nrp-1^−^ Tregs are shown in **f** alongside representative FACS plots. Immunofluorescence microscopy of EPX-positive eosinophils and of Foxp3^+^ Tregs in the gastric mucosa of infected mice is shown in **g** for two representative WT mice along with high magnification insets (scale bar, 10 μm). Treg vs. eosinophil numbers per mm^2^ are plotted for all mice in **h**; means of Treg numbers ± standard deviation/mm^2^ and the means ± standard deviation of Tregs/mm^2^ in direct contact with eosinophils are plotted in **i**. Two independent studies were pooled in **d**–**f**, and one study is shown in **h** and **i**. **j**, **k** PHIL mice and their WT littermates were infected with *H. pylori* strain PMSS1 for 6 weeks. Absolute counts of gastric lamina propria Foxp3^+^ Tregs and RORγt^+^ Nrp-1^−^ Tregs are shown relative to uninfected controls. Data in **j** and **k** are pooled from two studies. Statistical comparisons were performed by Mann–Whitney (two groups) or Kruskal–Wallis (more than two groups) test followed by Dunn’s post hoc test. *p < 0.05, **p < 0.01, ***p < 0.001, ****p < 0.0001, ns not significant.

Having shown earlier that eosinophils have an important immunoregulatory function in settings of chronic infection with *H. pylori*^10^, we aimed to examine how the conditional ablation of this lineage would affect the Treg compartment. To characterize the infection-induced gastric Treg compartment at high resolution, we first established a spectral flow cytometry panel for lymphocyte markers, among which 15 were selected because they are known to be expressed on Tregs (Supplementary Fig. 1a). The expression of these 15 markers clearly segregated Foxp3^+^ Tregs from uninfected and infected mice (Supplementary Fig. 1b). Three markers, i.e., Nrp-1, RORγt and ICOS were strongly differentially expressed by Foxp3^+^ Tregs from infected relative to uninfected mice, which allowed us to define five populations of gastric Tregs: (1) ICOS^−^ Nrp-1^+^ Tregs that are present already at steady state and do not respond to infection, (2) ICOS^int^ Nrp-1^int^ Tregs that are present at steady state, but respond to infection by upregulating ICOS, CD39 and CTLA4, (3) ICOS^high^ RORγt^+^ Nrp-1^−^ Tregs that appear de novo in the infection setting and express high levels of CD39, CD73 and CTLA4, (4) a subpopulation of 3 that expresses the proliferation marker Ki67 and (5) a subpopulation of population 3 that fails to express ICOS (Fig. 1a–c and Supplementary Fig. 1c). Only the three latter RORγt^+^ Nrp-1^−^ populations increase in number and/or frequency as a consequence of infection (Fig. 1b, c).

To examine how the conditional ablation of the eosinophil lineage would affect the Treg compartment, mice expressing *Cre* recombinase under the eosinophil peroxidase (EPO) promoter (Eo-*Cre*)^13^ were crossed with diphtheria toxin receptor (Dtr) transgenic mice, allowing for the specific and conditional depletion of eosinophils in the composite (Eo-*Cre*^Dtr^) strain. As previously reported^10^, biweekly DT administration resulted in a reduction of eosinophils in the gastric lamina propria by >80% and led to enhanced Th1 and CD8^+^ T-cell responses and improved immune control of *H. pylori* (Supplementary Fig. 1d–i). Although the recruitment of CD4^+^ T-cells, and of Foxp3^−^ conventional CD4^+^ T-cells was not generally affected by eosinophil depletion, we observed a significant reduction in Foxp3^+^ Treg frequencies (data not shown) and absolute numbers that affected Nrp-1-negative more than Nrp-1-positive Treg subsets (Fig. 1d, e and Supplementary Fig. 1j, k), and especially the RORγt^+^ Nrp-1^−^ Tregs described above (Fig. 1f). In the stomach, RORγt^+^ Nrp-1^−^ Tregs are positive for T-bet (Supplementary Fig. 1l).

To address whether eosinophils and Tregs co-localize in the gastric mucosa, we stained gastric sections of Eo-*Cre*^Dtr^ and their WT littermates for EPO expression (in red, Fig. 1g) and Foxp3 (in turquoise); both populations were found in close proximity to one another at both the base and the tip of gastric glands. Eosinophil and Treg numbers were positively correlated in WT mice, and Tregs were reduced—also as quantified by automated microscopy—upon eosinophil depletion (Fig. 1h, i). The phenotype of eosinophil-depleted Eo-*Cre*^Dtr^ mice was recapitulated in PHIL mice, which transgenically express diphtheria toxin (DT) under the EPO promoter and therefore constitutively lack eosinophils in all lymphoid and non-lymphoid tissues^13^. Similar to eosinophil-depleted Eo-*Cre*^Dtr^ mice, PHIL mice exhibited reduced *H. pylori* colonization levels (Supplementary Fig. 1m), and reduced numbers of Foxp3^+^ Tregs and RORγt^+^ Nrp-1^−^ Tregs despite normal CD4^+^ T-cell counts (Fig. 1j, k and Supplementary Fig. 1n, o). As the de novo generation of Tregs from naïve CD4^+^ T-cells takes place in the draining lymph nodes, which in the case of the gastric mucosa are the mesenteric lymph nodes (MLNs)^14,15^, we examined MLN Treg populations upon *H. pylori* infection but found no differences in this lymphoid organ (Supplementary Fig. 1p). The combined data suggest that eosinophils are dispensable for Treg priming in lymph nodes, but contribute either to the recruitment to, or local expansion of Tregs in infected tissues.

### Experimentally induced eosinophilia results in excessive Treg numbers

**Fig. 2:**
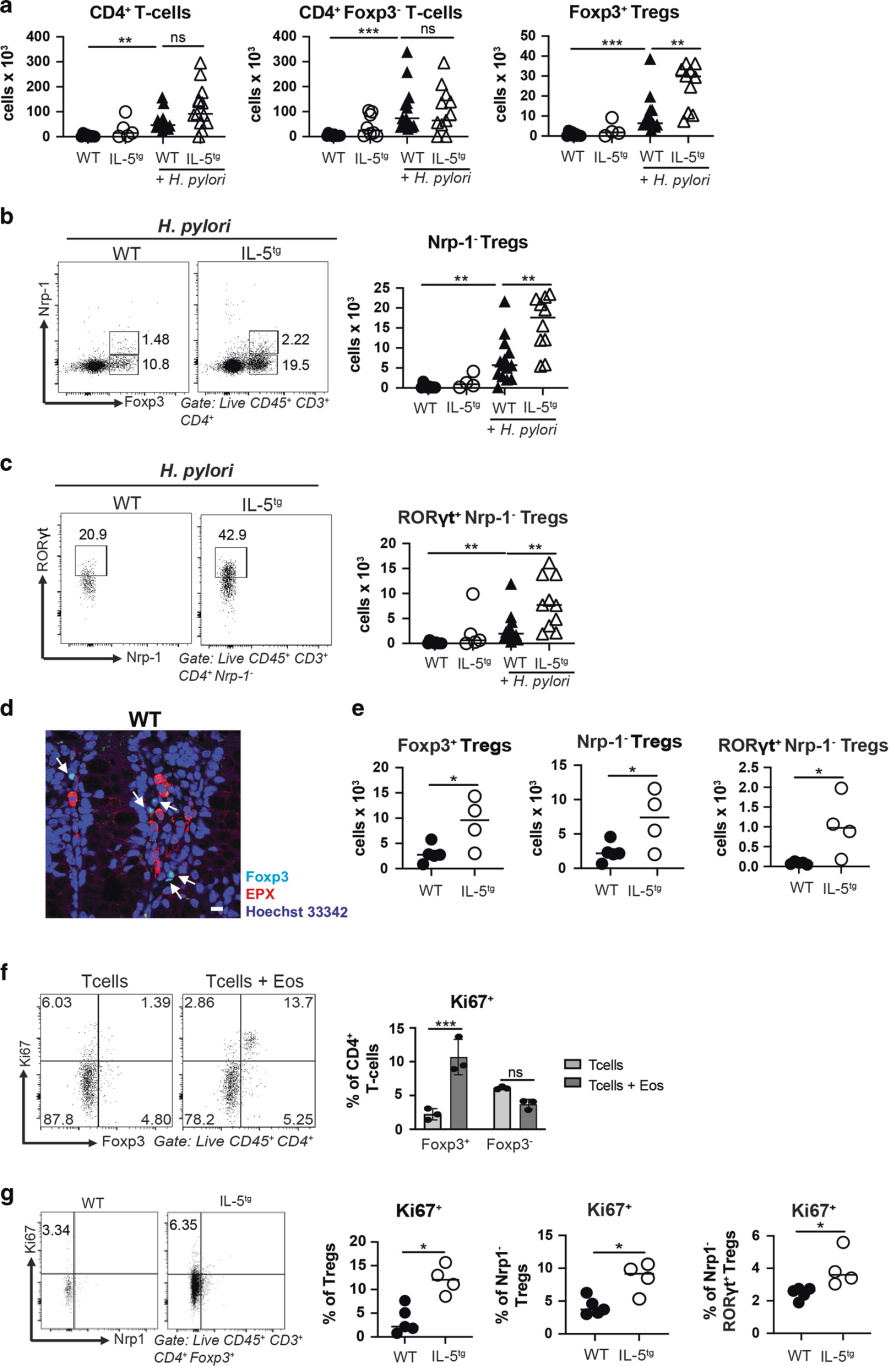
IL-5-driven eosinophilia promotes Treg expansion in infected tissues and the steady state colon. **a**–**d** IL-5-transgenic (IL-5^tg^) mice and their wild-type littermates were infected with *H. pylori* strain PMSS1 for 6 weeks. **a** Absolute counts of all gastric lamina propria CD4^+^ T-cells, CD4^+^ Foxp3^−^ conventional T-cells and CD4^+^ Foxp3^+^ Tregs, of infected IL-5^tg^ mice and WT littermates relative to uninfected age-matched controls. **b**, **c** Absolute counts of gastric Foxp3^+^ Nrp-1^−^ Tregs and RORγt^+^ Nrp-1^−^ Tregs of the mice shown in **a**; summary plots are shown alongside representative FACS plots of the two infected groups. Data in **a**–**c** are pooled from two independent studies. **d**, **e** IL-5^tg^ mice and their wild-type littermates were subjected to immunofluorescence microscopy and colonic lamina propria leukocyte isolation followed by flow cytometry. **d** Representative microscopic image of EPX-positive eosinophils and of Foxp3-positive Tregs in the colonic steady state mucosa of a WT mouse (scale bar: 10 μm). White arrows point to eosinophils and Tregs in close proximity. **e** Absolute counts of colonic Foxp3^+^ Tregs, Nrp-1^−^ Tregs and RORγt^+^ Nrp-1^−^ Tregs. **f** Splenic eosinophils immunomagnetically sorted from IL-5^tg^ mice were co-cultured with naïve splenic CD4^+^ T-cells from WT mice for 3 days at a 1:1 ratio; Ki67 expression were quantified by flow cytometry at the study endpoint. Representative FACS plots are shown alongside summary plots for two pooled studies. **g** Ki67 staining of the mice shown in **e**. Summary plots of the frequencies of Ki67^+^ among all Tregs, among Nrp-1^−^ Tregs and among RORγt^+^ Nrp-1^−^ Tregs are shown alongside representative FACS plots for Tregs. Statistical comparisons were performed by Mann–Whitney (two groups) or Kruskal–Wallis (more than two groups) test followed by Dunn’s post hoc test. *p < 0.05, **p < 0.01, ***p < 0.001, ****p < 0.0001, ns not significant.

We next asked whether the excessive production of eosinophils in a mouse strain that transgenically expresses IL-5 in CD4^+^ T-cells would result in aberrant Treg numbers at steady state or during infection. As reported previously, IL-5-transgenic mice are overtly normal and healthy in their first 6 months of life at least in the absence of helminth or allergen challenge^16^; this is also true if the line is maintained in our facility. IL-5-transgenic mice harbor at least five times more eosinophils not only in the blood as published previously^17^, but also in the *H. pylori*-infected gastric mucosa and the MLNs (Supplementary Fig. 2a–c). Eosinophils in IL-5-transgenic mice are more activated than their counterparts in wild-type littermates as judged by their surface Siglec-F and CD11b expression, whereas antibody-mediated IL-5 neutralization reduced the activation state of eosinophils (Supplementary Fig. 2d, e). IL-5-transgenic mice exhibited an overabundance of Tregs upon infection, despite harboring normal overall CD4^+^ T-cell and conventional CD4^+^ Foxp3^−^ T-cell counts (Fig. 2a). Among Tregs, Nrp-1^−^ Tregs and especially the subsets of RORγt^+^ Nrp-1^−^ Tregs and T-bet^+^ RORγt^+^ Nrp-1^−^ Tregs were strongly overrepresented, whereas Nrp-1-positive thymus-derived Tregs were present at wild-type levels (Fig. 2b, c and Supplementary Fig. 2f, g). *H. pylori* colonization levels and *H. pylori*-specific Th1 responses were not affected by IL-5-driven eosinophilia (Supplementary Fig. 2h, i). Interestingly, the MLNs of IL-5-transgenic mice -in contrast to those of WT mice- harbor a large population of eosinophils (Supplementary Fig. 2c), and this correlates with an increase in the MLN pool of RORγt^+^ Nrp-1^−^ and especially RORγt^+^ T-bet^+^ Nrp-1^−^, but not Nrp-1^+^ Tregs relative to wild-type littermates (Supplementary Fig. 2j).

To examine whether the absence or excessive presence of eosinophils in a tissue with a large steady state population of Tregs affects Treg numbers, we compared the colonic Treg populations of PHIL and IL-5-transgenic mice and their wild-type littermates. In the colon as in the infected stomach, Tregs were often found in close proximity to eosinophils as judged by Foxp3- and EPO-specific staining of colon sections (Fig. 2d). Whereas the overall CD4^+^ T-cell population, and the pool of conventional CD4^+^ T-cells not expressing Foxp3 were similar in PHIL and IL-5-transgenic mice and their respective littermates, the numbers of Foxp3^+^ Tregs differed substantially and correlated positively with the numbers of eosinophils (Fig. 2e and Supplementary Fig. 2k). As observed in the *H. pylori*-infected gastric mucosa, Nrp-1^−^ Tregs and their subpopulations were most strongly affected by the absence or excessive presence of eosinophils (Fig. 2e and Supplementary Fig. 2k).

To examine the effects of eosinophils on naïve T-cells and Tregs in vitro, we co-cultured immunomagnetically isolated, splenic eosinophils (purity: >90%; Supplementary Fig. 2l) derived from IL-5-transgenic mice with naïve splenic T-cells at a 1:1 ratio for 3 days and measured their Foxp3 expression and proliferation. As reported previously^18^, eosinophils induced Foxp3 expression in co-cultured naïve T-cells, although the effects were modest compared to the effects of recombinantly added TGF-β (Supplementary Fig. 2m). The induction of Foxp3 expression was observed irrespective of whether naïve T-cells were immunomagnetically or flow cytometrically sorted, and the separation of eosinophils and T-cells by a trans-well filter abrogated Foxp3 induction (Supplementary Fig. 2m, n). De novo generated Foxp3^+^ Tregs in co-culture with eosinophils, but not their Foxp3^−^ counterparts in the same culture, proliferated measurably as determined by Ki67 staining (Fig. 2f). Foxp3^+^ Tregs FACS-sorted from Foxp3^YFP^ reporter mice also proliferated upon co-culture with eosinophils, which could be reduced by neutralization of TGF-β (Supplementary Fig. 2o). The in vitro findings were confirmed by Ki67 staining of Tregs in vivo, as Ki67^+^ Tregs were more frequent in the colons of IL-5-transgenic mice than the colons of their wild-type littermates; the fraction of Ki67^+^ cells increased among Tregs, Nrp-1^−^ Tregs and RORγt^+^ Nrp-1^−^ Tregs (Fig. 2g). In conclusion, experimental eosinophilia due to transgenic IL-5 expression results in an overabundance of Tregs, and especially of peripherally induced Tregs expressing RORγt and T-bet; eosinophils appear to stimulate Treg, but not conventional T-cell expansion both in vitro and in vivo.

### Eosinophil-derived TGF-β is required for Treg expansion in the infected gastric and colonic mucosa

**Fig. 3:**
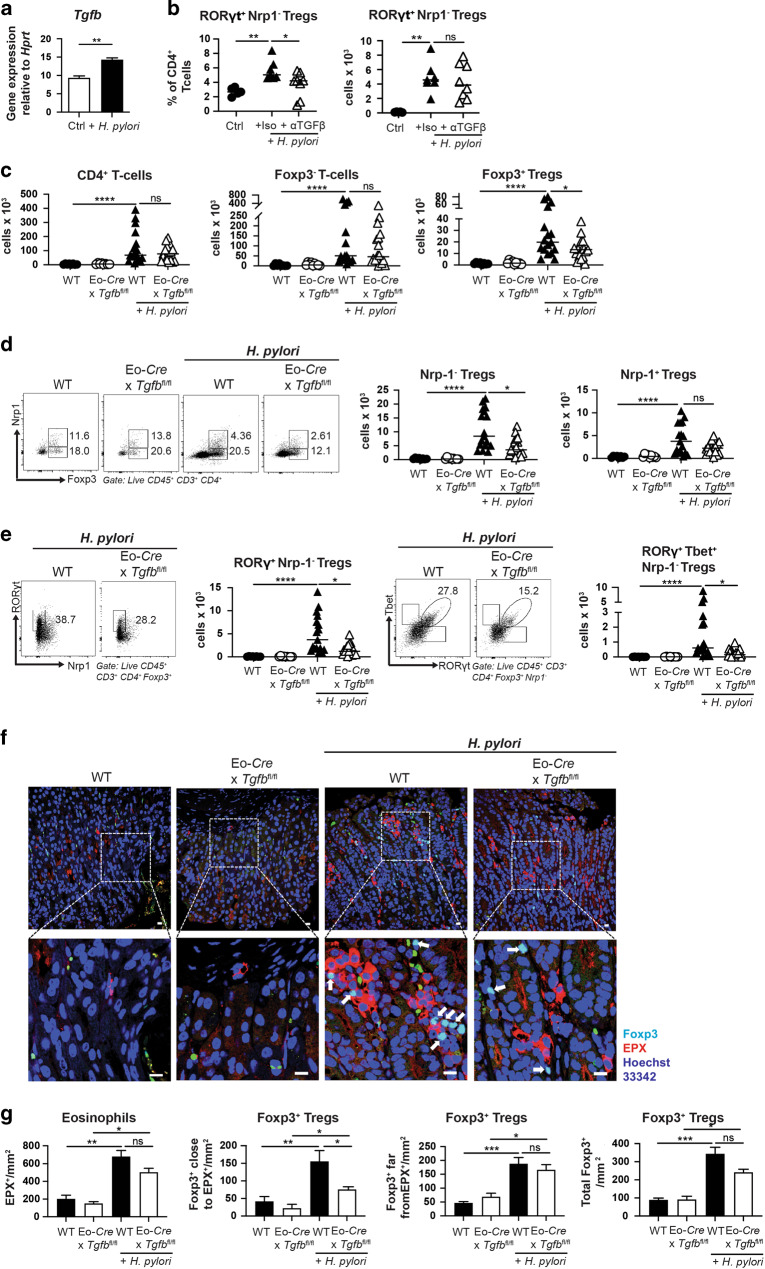
TGF-β production by eosinophils is required for Treg accumulation in gastrointestinal tissues during chronic infection with *H. pylori* and *H. hepaticus*. **a** Eosinophils sorted from the spleens of IL-5^tg^ mice were exposed to live *H. pylori* PMSS1 at a multiplicity of infection of 10 for 6 h, and subjected to *Tgfb*-specific qRT-PCR. **b** Wild-type C57/BL6 mice were infected with *H. pylori* strain PMSS1 for 6 weeks, and treated with twice-weekly doses of 250 μg anti-TGF-β neutralizing or isotype control antibody. The frequencies of RORγt^+^ Nrp-1^−^ Tregs among CD4^+^ T-cells, and their absolute counts are shown. Data are from one representative study of two. **c**–**e** *Eo*-Cre x *Tgfb*^fl/fl^ mice and their wild-type littermates were infected with *H. pylori* strain PMSS1 for 6 weeks. Absolute counts of all gastric lamina propria CD4^+^ T-cells, CD4^+^ Foxp3^−^ conventional T-cells and CD4^+^ Foxp3^+^ Tregs are shown in **c** of infected *Eo*-Cre x *Tgfb*^fl/fl^ mice and WT littermates relative to uninfected age-matched controls. Absolute counts of gastric Foxp3^+^ Nrp-1^−^ Tregs, Foxp3^+^ Nrp-1^+^ Tregs, RORγt^+^ Nrp-1^−^ Tregs and RORγt^+^ T-bet^+^ Nrp-1^−^ Tregs of the same mice are shown in **d** and **e** alongside representative FACS plots. Data in **c**–**e** are pooled from two independent studies. **f**, **g** Immunofluorescence microscopy of EPX-positive eosinophils and of Foxp-3-positive Tregs in the gastric mucosa of *H. pylori*-infected and uninfected mice of the indicated genotypes (scale bar: 10 μm). White arrows point to Foxp3^+^ Tregs. The quantification of eosinophils, of Foxp3^+^ Tregs in close proximity, of Foxp3^+^ Tregs distant from eosinophils, and of all Tregs, all per mm^2^, as determined by automated quantification, are shown for 5–7 mice per group in **g**. Statistical comparisons were performed by Mann–Whitney (two groups) or Kruskal–Wallis (more than two groups) test followed by Dunn’s post hoc test. *p < 0.05, **p < 0.01, ***p < 0.001, ****p < 0.0001, ns not significant.

Eosinophils exposed to live bacteria in vitro or in vivo express numerous cytokines, chemokines and transcription factors with both immunostimulatory and immunoregulatory activities^10^. As TGF-β plays a critical role in gut Treg biology, driving and stabilizing Foxp3 expression through the binding of SMAD3 to the *Foxp3* enhancer^19–21^, we hypothesized that TGF-β produced by eosinophils might support Treg generation or recruitment to infected tissues. To test this idea, we first assessed TGF-β production by eosinophils in response to *H. pylori*. Indeed, even a short exposure of splenic eosinophils derived from IL-5-transgenic mice to live *H. pylori* was sufficient to induce TGF-β expression (Fig. 3a). The neutralization of TGF-β by a suitable antibody during the entire four-week time course of an *H. pylori* infection in vivo decreased the frequency of RORγt^+^ Nrp-1^−^ Tregs in the gastric lamina propria, but failed to affect the absolute counts (Fig. 3b). To address the contribution of eosinophil-intrinsic TGF-β production to Treg accumulation upon infection, we crossed *Tgfb*^fl/fl^ and Eo-*Cre* mice to produce a strain lacking TGF-β production specifically in the eosinophil compartment. Whereas the residence of CD4^+^ T-cells and of CD4^+^ Foxp3^−^ conventional T-cells in the gastric lamina propria was not impaired by the conditional ablation of *Tgfb* in eosinophils, we detected a clear reduction in Tregs (Fig. 3c), in peripherally induced Nrp-1^−^ Tregs and their RORγt^+^ and RORγt^+^ T-bet^+^ subpopulations (Fig. 3d, e); the Nrp-1^+^ Treg population was not significantly reduced (Fig. 3d). The numbers and frequencies of eosinophils in *Tgfb*^fl/fl^ x Eo-*Cre* mice were normal in the examined tissues, both at steady state and during infection (Supplementary Fig. 3a). Interestingly, the co-localization of eosinophils and Tregs in clusters upon *H. pylori* infection depends at least in part on TGF-β production by eosinophils (Fig. 3f, g). As observed for Eo-*Cre*^DTR^ mice, the reduction in de novo-induced Treg populations was specific to the infected gastric tissue, and not observed in the MLNs (Supplementary Fig. 3b). The loss of TGF-β signaling in eosinophils did not affect bacterial infection control in a major way, as Th1, Th17 and CD8^+^ T-cell responses were similar and we detected only non-significant trends toward lower *H. pylori* colonization in *Tgfb*^fl/fl^ x Eo-*Cre* mice (Supplementary Fig. 3c, d). Spectral flow cytometry revealed that, among the five identified gastric Treg populations introduced in Fig. 1, only the three RORγt^+^ Nrp-1^−^ populations (3–5) differed in absolute numbers as a function of *Tgfb* ablation in eosinophils (Supplementary Fig. 3e). As TGF-β has been implicated in IgA class switching and IgA production in mice, and in murine as well as human cell cultures^22–24^, and the extent of IgA coating of microbes has been shown to modulate the differentiation of intestinal RORγt^+^ Tregs^25^, we set out to investigate a possible eosinophil/TGF-β/IgA/Treg axis in our experimental system. We indeed found that the induction of a strong gastric IgA response to *H. pylori* was dependent on TGF-β production by eosinophils (Supplementary Fig. 3f), suggesting that eosinophils may serve as a relevant source of the TGF-β that promotes IgA production in the stomach. However, infection of IgA-deficient mice with *H. pylori* led to similar gastric absolute numbers of Foxp3^+^ Tregs, Nrp-1^-^ Tregs and RORγt^+^ Nrp-1^–^ Tregs as the infection of wild-type controls (Supplementary Fig. 3g), indicating that, at least in the context of this bacterial infection, Treg populations are neither negatively nor positively regulated by IgA. We also examined whether the expression of TGF-β by other gastric leukocyte populations would be affected by the loss of TGF-β in the eosinophil compartment; this was not the case, as staining for latent protein LAP, to which TGF-β is bound and from which it has to be released in its active form, revealed similar LAP/TGF-β levels in macrophages, neutrophils, CD11b^+^ DCs and CD103^+^ DCs irrespective of the TGF-β proficiency of eosinophils (Supplementary Fig. 3h). This analysis also uncovered that eosinophils in the gastric mucosa are neither the most numerically abundant, nor -on a per cell basis- the most productive source of TGF-β.

To address whether eosinophil-derived TGF-β might be required not only for gastric but also for colonic Treg accumulation upon bacterial infection, we infected *Tgfb*^fl/fl^ x Eo-*Cre* mice with the intestinal pathobiont *H. hepaticus*. Just like *H. pylori*, *H. hepaticus* induced the accumulation of Tregs, and especially of RORγt^+^ Nrp-1^−^ Tregs in the colonic mucosa, which was dependent on eosinophil-derived TGF-β (Supplementary Fig. 3i). *Tgfb*^fl/fl^ x Eo-*Cre* mice exhibited lower *H. hepaticus* colonization than their wild-type littermates, but showed no changes in their CD4^+^ effector T-cell response (Supplementary Fig. 3j). The combined data suggest that the generation of a tissue-resident pool of Tregs requires eosinophil-derived TGF-β during GI bacterial infection.

### Eosinophil-derived TGF-β is required for the tissue residence of de novo-induced Tregs during acute bacterial infection as well as allergen challenge

**Fig. 4:**
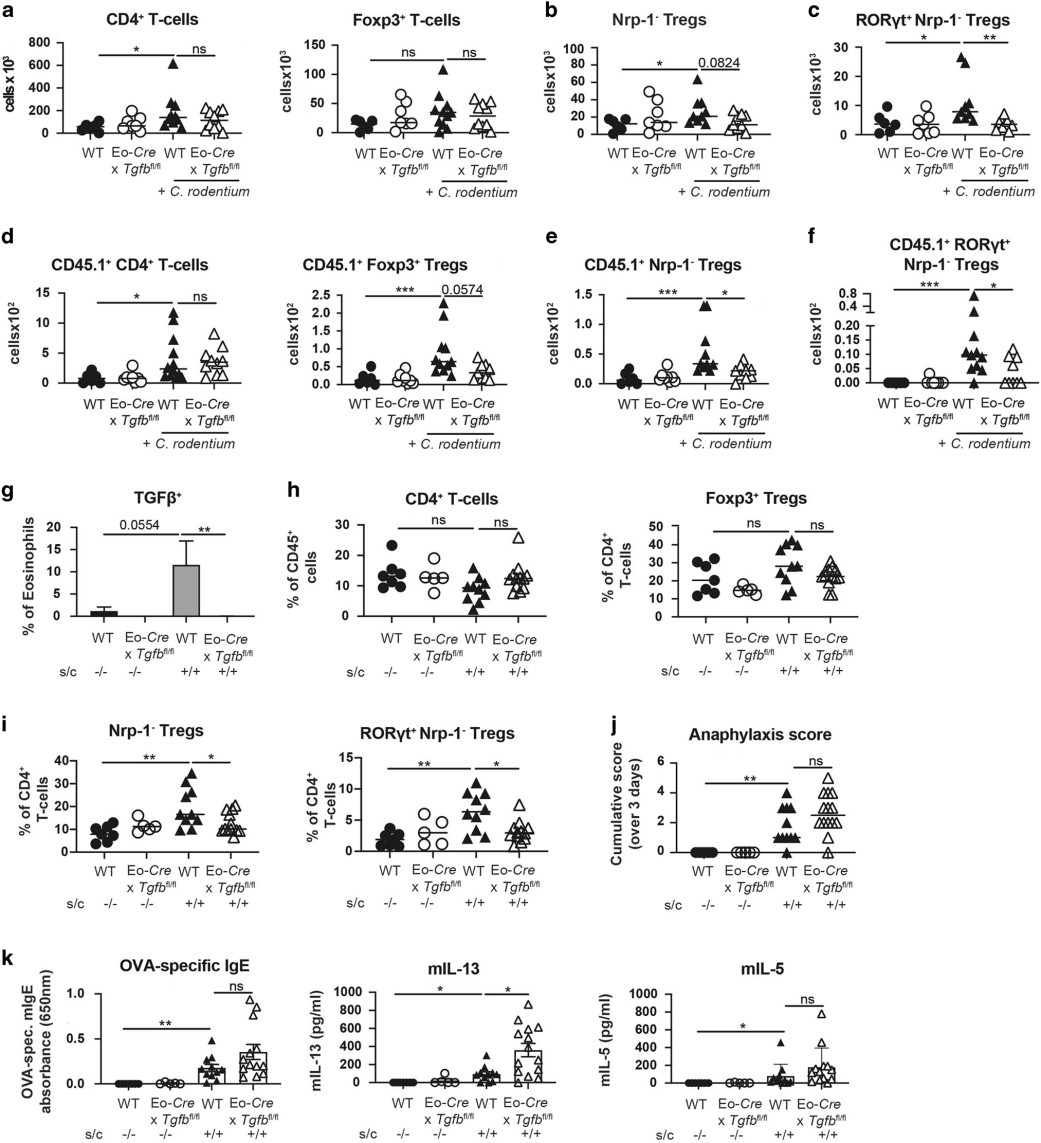
TGF-β production by eosinophils is required for Treg accumulation during acute infection with *C. rodentium* and during allergen challenge. **a**–**c** *Eo*-Cre x *Tgfb*^fl/fl^ mice and their wild-type littermates were infected with *C. rodentium* for 12 days. Absolute counts per organ of all colonic lamina propria CD4^+^ T-cells and CD4^+^ Foxp3^+^ Tregs are shown in **a**, and absolute counts per organ of colonic Foxp3^+^ Nrp-1^−^ Tregs and of RORγt^+^ Nrp-1^−^ Tregs are shown in **b** and **c** relative to uninfected age-matched controls. Data in **a**–**c** are pooled from two independent studies. **d**–**f** *Eo*-Cre x *Tgfb*^fl/fl^ mice and their wild-type littermates were infected with *C. rodentium* for 12 days, and additionally injected with CD45.1^+^ OT-II splenocytes and one intravenous dose of OVA peptide 7 days prior to the termination of the study. Absolute counts per organ of the indicated adoptively transferred CD45.1^+^ CD4^+^ T-cell and Treg populations are shown in **d**–**f**. Data in **d**–**f** are pooled from two independent studies. **g**–**k** *Eo*-Cre x *Tgfb*^fl/fl^ mice and their wild-type littermates were intraperitoneally sensitized twice with alum-adjuvanted ovalbumin at 5 and 7 weeks of age and challenged on four consecutive days with orally administered ovalbumin 4 weeks after the first sensitization (s/c). Control mice were mock-sensitized and -challenged with PBS only. TGF-β production by colonic eosinophils as determined by flow cytometry is shown in **g**; frequencies of CD4^+^ T-cells among all colonic CD45^+^ cells and of Foxp3^+^ Tregs among all CD4^+^ T-cells are shown in **h**. The frequencies of colonic Foxp3^+^ Nrp-1^−^ Tregs and of RORγt^+^ Nrp-1^−^ Tregs among CD4^+^ T-cells are shown in **i**. Cumulative anaphylaxis score over 3 days are shown in **j**, and OVA-specific IgE in serum, and IL-13 and IL-5 production by OVA-restimulated splenocytes, as assessed by ELISA, is shown in **k**. Data from two independent studies are pooled in **g**–**k**. Statistical comparisons were performed by Kruskal–Wallis test followed by Dunn’s post hoc test. *p < 0.05, **p < 0.01, ***p < 0.001, ****p < 0.0001, ns not significant.

To address whether eosinophil-derived TGF-β contributes to the generation of a de novo-arising Treg pool also in other bacterial challenge or allergen challenge models, we first turned to a model of *Citrobacter rodentium* infection. *C. rodentium* colonizes the cecum and colon and may additionally disseminate to the MLNs and the spleen. The control of *C. rodentium* requires a strong, mixed Th1/Th17 response and ultimately the production of antimicrobial peptides^26^. Exposure of cultured eosinophils to live *C. rodentium* induced strong expression of TGF-β (Supplementary Fig. 4a). Whereas the effector branches of the immune response to *C. rodentium*, and the colonization levels of the bacteria, were not measurably affected by the eosinophil-specific loss of TGF-β (except for the colonization of the cecum; Supplementary Fig. 4b–d), the colonic Treg compartment showed similar trends as observed for *H. pylori* and *H. hepaticus*. RORγt^+^ Nrp-1^−^ Foxp3^+^ Tregs were induced by infection with *C. rodentium* in wild-type littermates, but not *Tgfb*^fl/fl^ x *Eo*-Cre mice; no substantial differences were seen relative to baseline in the overall colonic Foxp3^+^ Treg and Nrp-1^−^ Foxp3^+^ Treg populations (Fig. 4a–c). Among RORγt^+^ Nrp-1^−^ Foxp3^+^ Tregs, the proliferating (Ki67^+^) fraction of the population was reduced as a consequence of TGF-β deficiency in the eosinophil compartment (Supplementary Fig. 4e), indicating that the local expansion of newly formed Tregs requires TGF-β. Interestingly, adoptively transferred OT-II T-cells that are transgenic for the MHCII-restricted chicken ovalbumin peptide OVA_323-339_, and that were injected together with their cognate peptide, recapitulated the differences observed in the endogenous Treg compartment. Transferred OT-II T-cells expressed Foxp3 and were recruited to the colon only in the presence of *C. rodentium*, and only in wild-type littermates; *Tgfb*^fl/fl^ x *Eo*-Cre mice lacked colonic Foxp3^+^ OT-II T-cells, and especially the RORγt^+^ Nrp-1^−^ subpopulation (Fig. 4d–f).

To investigate the consequences of TGF-β deficiency in the eosinophil compartment on Treg induction in a setting of sterile inflammation, we turned to a model of allergic sensitization and challenge that we had shown previously to be accompanied by colonic Treg accumulation^27^. We used intraperitoneal challenge with alum-adjuvanted ovalbumin, followed by oral challenge with ovalbumin as this regimen proved to be robust and comparatively well-tolerated in a previously conducted comparative study of four different food allergy settings^28^. Allergen sensitization and challenge induced TGF-β expression by eosinophils (Fig. 4g). In this model, as in the bacterial infection models, colonic Treg accumulation depended on allergen sensitization and challenge on the one hand, and eosinophil-derived TGF-β on the other; RORγt^+^ Nrp-1^−^ Tregs were most affected by the genetic ablation of eosinophil-derived TGF-β (Fig. 4h, i). Ovalbumin sensitization and challenge resulted in anaphylactic reactions to the challenge, splenic production of the Th2 cytokines IL-5 and IL-13, and strongly enhanced levels of ovalbumin-specific, circulating IgE; IL-13 levels were higher, but none of the other allergy parameters were different in challenged *Tgfb*^fl/fl^ x *Eo*-Cre mice relative to their wild-type littermates (Fig. 4j, k). House dust mite-induced allergic airway hyper-responsiveness was more severe in *Tgfb*^fl/fl^ x *Eo*-Cre mice than their wild-type littermates as judged by the quantification of total leukocytes and of eosinophils in the bronchoalveolar lavage fluid (Supplementary Fig. 4f). The combined data suggest that eosinophil-derived TGF-β contributes to the generation of a tissue-resident population of peripherally/de novo-induced Tregs not only during bacterial infection, but also during responses to orally administered antigens. In some but not all models, eosinophil-derived TGF-β is required to limit the immunopathological consequences of unrestricted effector T-cell activation.

In this study, we have examined the contribution of eosinophils, and/or of eosinophil-derived TGF-β in three settings in which peripheral Tregs (pTregs) arise de novo and are known to play critical roles in maintaining immune tolerance and tissue homeostasis, i.e., (1) the steady state colon, in which pTregs sustain immune tolerance toward highly abundant but harmless commensal and food-borne antigens^29^, (2) an oral tolerance model in which repeated ovalbumin challenge results in Th2-polarized responses that are accompanied—and counteracted—by de novo-induced Tregs^30^, and (3) three bacterial infection models in which a mixed Th1/Th17 response is balanced by de novo-induced Tregs^14,31^. The results point to an indispensable role of eosinophils and eosinophil-derived TGF-β during the second, but not during the first phase of the two-step process of Nrp-1^−^ Treg cell differentiation and tissue homing. The first step, i.e., the conversion of naïve CD4^+^ T-cells to Foxp3-expressing Tregs, most efficiently takes place in the gut-draining MLNs^32,33^, is mainly driven by dendritic cells (DCs) loaded with gut-derived antigens, and depends on retinoic acid and DC-derived TGF-β^33–35^. This process is not affected by eosinophils. Rather, we uncover a critical contribution of eosinophils to the second phase of pTreg tissue homing and expansion. In this second phase, recently converted Tregs migrate to the GI lamina propria, re-encounter their cognate antigens and undergo further differentiation and expansion in a process that is known to be propelled by intestinal macrophages^36,37^. Our results suggest that eosinophils, possibly via their direct proximity to Tregs, boost the proliferation and expansion of Tregs in a TGF-β-dependent manner; this effect appears to be rather specific to recently generated de novo-induced Nrp-1^-^ Tregs, and does not apply to the same extent to conventional T-cells or thymic Tregs. We investigated whether the genetic ablation of TGF-β signaling in macrophages phenocopies the effects of TGF-β loss in eosinophils by infecting *Tgfbr2*^fl^ x LysM-*Cre* mice with *H. pylori*, but found no evidence to support this idea (data not shown). The excessive production of eosinophils by IL-5-transgenic mice is sufficient to generate an overabundance of pTregs, with pTreg numbers increasing proportionally to eosinophil numbers in GI tissues, and in lymph nodes of IL-5-transgenic mice.

pTreg deficiency induced by genetic ablation of the *Foxp3* intronic enhancer element CNS1^19^ has mostly been linked to age-dependent mucosal type 2 pathologies^38^ and to dysregulated innate and adaptive type 2 responses to microbial exposure^29^. Functional pTreg defects resulting from *Rorc* (encoding RORγt) ablation in Tregs, or from early-life exposure to antibiotics associated with dysbiosis, also lead to enhanced susceptibility to food allergy and other types of type 2 pathologies^27,30^. Our data from mice defective for TGF-β production by eosinophils, which lack pTregs but have normal complements of eosinophils in all examined tissues, lend further support to the previously proposed idea that pTregs control type 2, but not type 1 responses^29,39^. Experimental evidence for the notion that intestinal RORγt^+^ Tregs predominantly control type 2 responses comes from models of oxazolone-induced, Th2 cytokine-driven colitis and of helminth infection, in which the conditional ablation of RORγt in Tregs results in more severe disease and improved helminth clearance, respectively^39^; the ILC2- and Th2 cell-specific production of the type 2 cytokines IL-4, IL-5 and IL-13 during assembly of a normal intestinal microbiota is also strongly elevated in the absence of RORγt^+^ Tregs^29^. Whereas we observed enhanced type 2 responses to ovalbumin and house dust mite allergen in *Tgfb*^fl^ x Eo-*Cre* mice, this was not the case for the type 1, or mixed type 1/3 responses that are typical of *Helicobacter* and *Citrobacter* infections. In contrast, the ablation of the entire eosinophil lineage results in overshooting Th1 responses and improved immune control of *Helicobacter* infections, as shown here and previously^10^, which could be attributed to direct, contact- and PD-1-dependent effects of eosinophils on Th1 cells. The direct suppressive effects of eosinophils on T-cells were dependent on the ability of eosinophils to sense IFN-γ, and to respond to IFN-γ conditioning by PD-1 upregulation^10^. The combined data indicate that eosinophils are conditioned by their microenvironment to prevent excessive T-cell responses and preserve tissue homeostasis through both direct (especially in type 1 settings) and indirect (type 2 settings) mechanisms.

## Methods

### Mice, pathogens and infections

B6-*Gt(ROSA)26Sortm1(Dtr)ThBu* (DTR) mice were described previously^40^. *Tgfb*^fl/fl^ mice (stock 010721) were purchased from the Jackson Laboratories. Both strains were crossed with mice expressing Cre under the EPO promoter (Eo-*Cre*)^41^, obtained from J.J. Lee (Mayo Clinic, Phoenix, AZ). Cre-negative littermates were used as controls throughout. Eosinophil-deficient mice (PHIL)^13^ were obtained from J.J. Lee (Mayo Clinic, Phoenix, AZ). IL-5–transgenic mice have been described previously^17^. Foxp^YFP^ reporter mice (B6.129(Cg)-Foxp3 < tm4(YFP/icre)Ayr > /J; stock 016959) were purchased from the Jackson Laboratories. All strains were bred and maintained under specific pathogen-free conditions in accredited animal facilities at the University of Zurich. Eo-*Cre*^DTR^ mice were treated with 15 ng/g body weight DT twice a week for 6 weeks for eosinophil depletion, initiated on the day of *H. pylori* infection. For in vivo neutralization of TGF-β (clone 1D11.16.8) or IL-5 (clone TRFK5, both BioXcell), mice received twice-weekly doses of 250 μg antibody. The *H. pylori* strain used in this study, PMSS1, is a clinical isolate of a patient with duodenal ulcer and the parental strain of the mouse-derivative Sydney strain 1 (SS1*)*^42^ and was originally obtained from Adrian Lee, Univ. of New South Wales, Sydney, Australia. *H. pylori* was grown on horse blood agar plates and in liquid culture as described previously^42^. Cultures were routinely assessed by light microscopy for contamination, morphology, and motility. Mice were infected orally on two consecutive days with 10^8^ CFU *H. pylori* PMSS1 at >6 weeks of age. To assess *H. pylori* colonization, gastric tissue was homogenized in Brucella broth and plated on horse blood agar plates supplemented antibiotics as described^42^. Colonies were counted after 5 days of culture at 37 °C under microaerophilic conditions. The nalidixic acid (NAL)-resistant *C. rodentium* strain ICC169 was grown overnight at 37 °C in Luria broth (LB) supplemented with NAL (50 μg/ml; Sigma). Mice were infected orally with 5 × 10^8^ bacteria for 12 days. To assess *C. rodentium* colonization, cecal, colonic and MLN tissues were homogenized in PBS, diluted, and plated on LB plates supplemented with NAL. Colonies were counted after 18 h of culture at 37 °C. Colonic and cecal bacterial loads were normalized to tissue weight. For the purpose of ovalbumin (OVA) sensitization and challenge, mice were sensitized twice intraperitoneally (i.p.) with 50 mg of OVA (Sigma; A5503-5G) emulsified in aluminum hydroxide (Imject alum, 77161; Thermo Scientific) on days 0 and 14, followed by challenge via oral gavage on days 28, 29, 30, and 31 with 60 mg of OVA. The readouts of the ovalbumin challenge model and the protocols for immunofluorescence staining of gastric and colonic tissue sections are described in detail in the Supplementary Methods, along with eosinophil/Treg co-culture protocols and *H. hepaticus* culture conditions and quantification. IgA was quantified by ELISA (Invitrogen). All animal experimentation was reviewed and approved by the Zurich Cantonal Veterinary Office (licenses ZH140/2017, ZH021/2020 and ZH086/2020) and adheres to the regulations of the Swiss National Veterinary Office.

### Leukocyte isolation and flow cytometry

For lamina propria leukocyte isolation, GI tissues were opened longitudinally, washed, and cut into pieces. Pieces were incubated in HBSS with 10% FCS and 5 mM EDTA at 37 °C to remove epithelial cells. Tissue was digested at 37 °C for 50 min in a shaking incubator with 15 mM HEPES, 500 U/ml of type IV collagenase (Sigma-Aldrich) and 500 U/ml of type VIII collagenase (Sigma-Aldrich), and 0.05 mg/ml DNase I in supplemented RPMI 1640 medium. Cells were then layered onto a 40/80% Percoll gradient and centrifuged, and the interface was washed in PBS with 0.5% BSA. For surface staining, cells were stained in PBS with 0.5% BSA with a fixable viability dye and a combination of the following antibodies: CD4 (RM4-5), CD45 (30-F11), L/D, CD3, and Nrp-1, CD11b (M1/70), CD11c (N418), F4/80 (BM8), Ly6G (1A8), MHC-II (M5/114.15.2), TGFb1 LAP (TW7–16B4), all from BioLegend and anti-mouse Siglec-F (E50-2440) from BD Biosciences. Fc block (anti-CD16/CD32, Affymetrix) was included to minimize nonspecific antibody binding. For intracellular cytokine staining of T-cells, cells were incubated for 3.5 h in complete IMDM containing 0.1 µM phorbol 12-myristate 13-acetate and 1 µM ionomycin with 1:1000 Brefeldin A (eBioscience) and GolgiStop solutions (BD Biosciences) at 37 °C in a humidified incubator with 5% CO_2_. Following surface staining, cells were fixed and permeabilized with the Cytofix/Cytoperm Fixation/Permeabilization Solution kit (BD Biosciences) according to the manufacturer’s instructions. Cells were then stained for 20 min with antibodies to TNF-α (TC11-18H10.1) and IFN-γ (XMG1.2), both from Biolegend. For the staining of transcription factors, cells were surface stained and fixed and permeabilized with the Foxp3/Transcription Factor Staining Buffer Set (eBioscience) according to the manufacturer’s instructions. Cells were then stained for 50 min with antibodies to mouse Foxp3 (MF-14, Biolegend), T-bet (4B10, Biolegend), Rorγt (Q31-378, BD Bioscience) or Ki67 (16A8, Biolegend). Total leukocyte counts were determined by adding countBright Absolute Counting Beads (Life Technologies) to each sample before analysis. Samples were analyzed on a LSRII Fortessa (BD Biosciences). Flow-cytometric analyses were performed using FlowJo software (Tree Star). The protocol for spectral flow cytometry, and the antibodies used are described in the Supplementary Methods.

### Statistics

Statistical analysis was performed with Prism 9.0 (GraphPad Software). The nonparametric Mann–Whitney *U* test was used for all direct statistical comparisons between two groups. Nonparametric one-way ANOVA (Kruskal–Wallis) was used for comparisons of more than two groups with Dunn’s multiple comparisons correction. Differences were considered statistically significant when *p* < 0.05: *p < 0.05, **p < 0.01, ***p < 0.001, and ****p < 0.0001.

## Supplementary information

supplemental figures and methods
