# Supplementary material for: TGF-β production by eosinophils drives the expansion of peripherally induced neuropilin− RORγt+ regulatory T-cells during bacterial and allergen challenge
Source: Mucosal Immunol. 2022 Feb 15;15(3):504–14. doi: 10.1038/s41385-022-00484-0 (PMC9038533; doi:10.1038/s41385-022-00484-0)
Supplement: Supplementary file 1 — supplemental figures and methods [file 41385_2022_484_MOESM1_ESM.pdf]

Supplementary Figures

Supplementary Figure 1

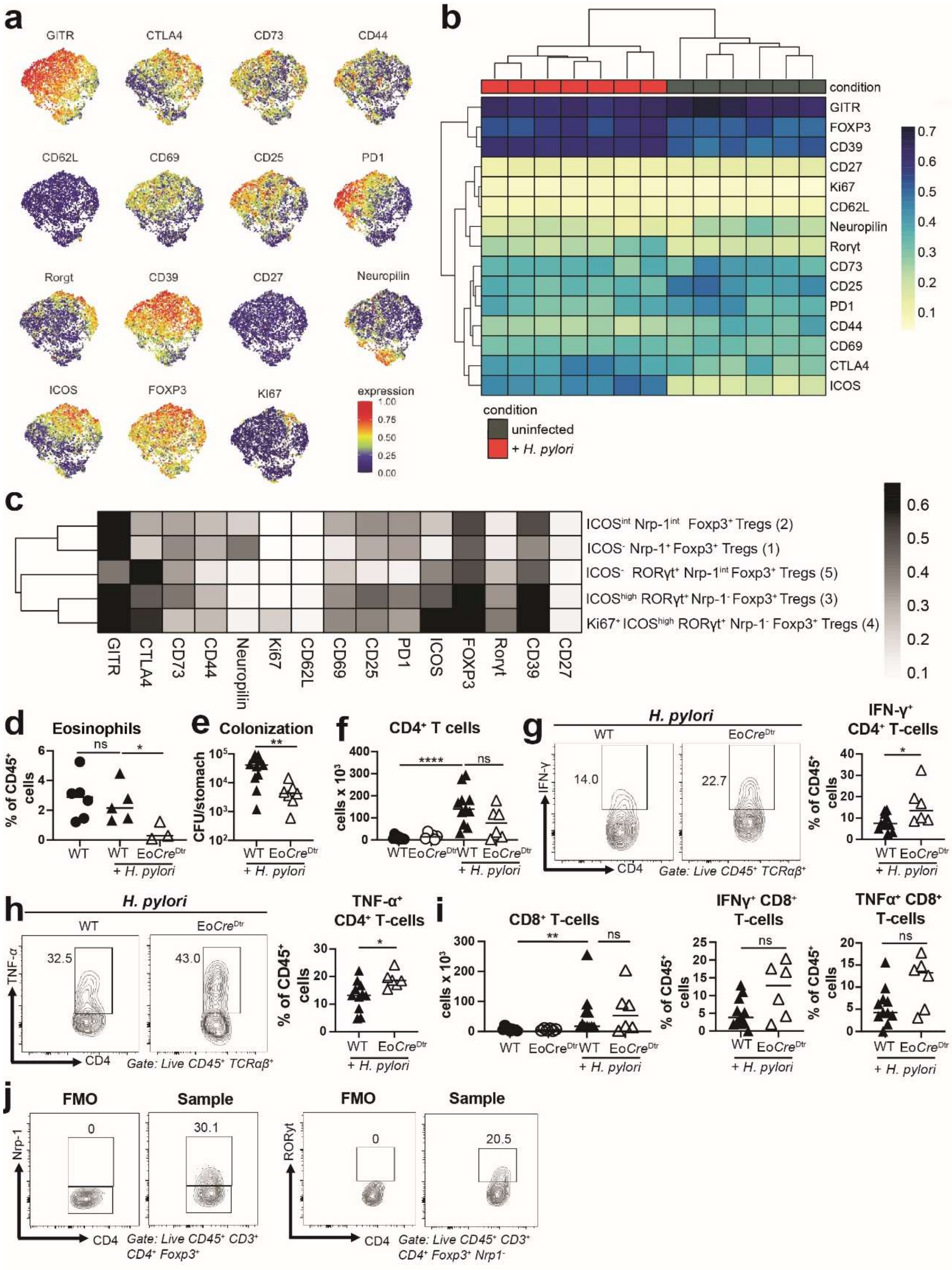

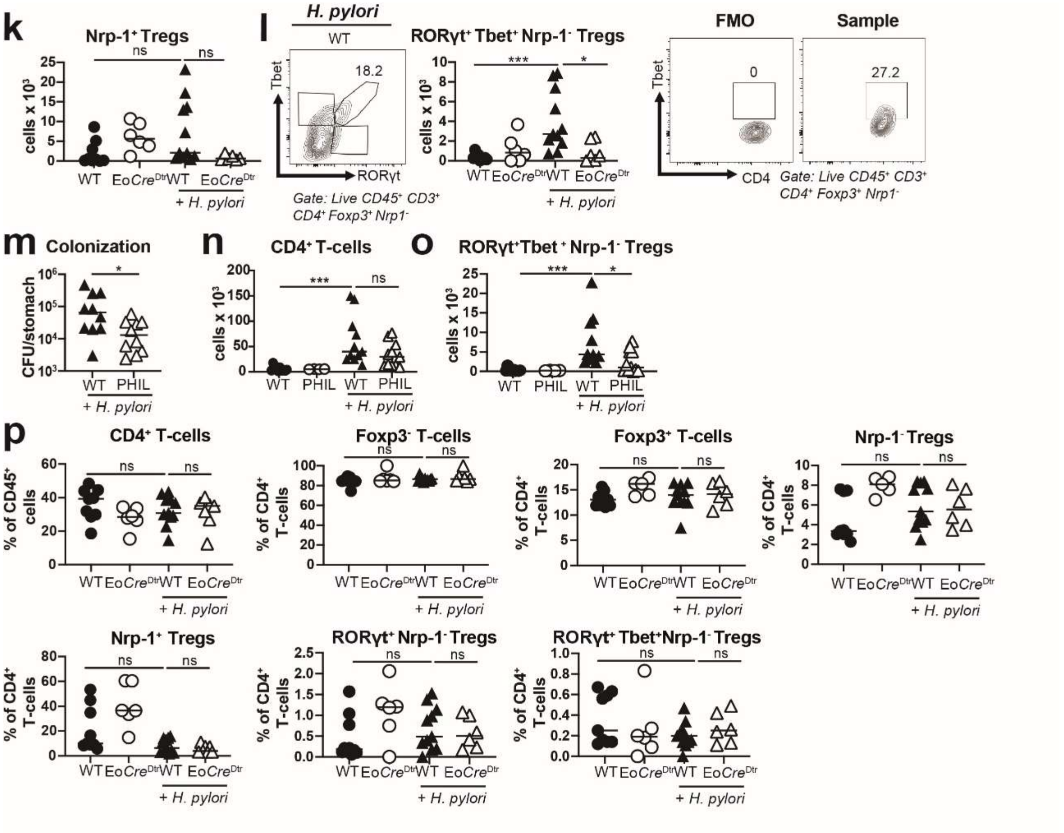

Suppl. Figure 1. Effector T-cell and Treg responses in the gastric mucosa differ as a function of *H. pylori* infection and eosinophil deficiency. a, UMAP showing the expression intensity of the indicated 15 markers on 1000 stochastically selected gastric CD4$^+$ Foxp3$^+$ Tregs from seven wild type mice that had been infected for 12 weeks with *H. pylori* as well as six age-matched littermates. **b**, Heatmap showing the expression of each of the 15 markers on CD4$^+$ Foxp3$^+$ Tregs of the same seven infected and six control mice as shown in a. **c,** Heat map showing the average expression of the 15 Treg markers by the five Treg populations of interest. **d-i,** Eo-*Cre*^{DTR} mice and their wild-type (Cre$^-$, WT) littermates were infected with *H. pylori* strain PMSS1 for six weeks; all mice (WT and Eo-*Cre*^{DTR}) received diphtheria toxin (DT) twice a week for the entire time course to deplete eosinophils. Frequencies of SiglecF$^+$ eosinophils among all gastric lamina propria leukocytes are shown in d. *H. pylori* colonization as determined by plating and colony counting is shown in e; CFU, colony forming units. Absolute counts of gastric lamina propria CD4$^+$ T-cells of infected Eo-*Cre*^{DTR} mice and WT littermates, relative to uninfected age-matched controls are presented in f. IFN-γ^+ and TNF-α^+ CD4$^+$ T-cells among all CD45$^+$ leukocytes, as determined by PMA/ionomycin restimulation and intracellular cytokine staining are presented in g and h; representative FACS plots are shown alongside summary plots for all mice. Absolute counts of gastric lamina propria CD8$^+$ T-cells and frequencies of IFN-γ^+ and TNF-α^+ CD8$^+$ T-cells among all CD45$^+$ leukocytes are shown in i of the same mice. **j,** Representative FACS plots demonstrating the specificity of the Nrp-1 antibody (left panels) and the RORγt antibody (right panels) used throughout in this work. FMO, fluorescence minus one. **k**, Absolute counts of gastric Foxp3$^+$ Nrp-1$^+$ Tregs of the mice shown in main Figure 1d-f. **l**, Absolute counts of gastric RORγt$^+$ Tbet$^+$ Nrp-1$^-$ Tregs of the mice shown in main Figure 1d-f are shown alongside a representative FACS plot of an *H. pylori*-infected WT mouse; the FACS plots (right panels) demonstrate the specificity of the Tbet antibody. **m-o,** PHIL mice and their WT littermates were infected with *H. pylori* strain PMSS1 for six weeks. The *H. pylori* colonization as determined by plating and colony counting is shown in m; absolute counts of

gastric lamina propria $CD4^+$ T-cells and of RORγt$^+$ Tbet$^+$ Nrp-1$^-$ Tregs of infected PHIL and WT mice are presented in n and o relative to uninfected controls. Data in e-i, k,l and m-o are pooled from two studies; data are d are from one representative study. **p,** Frequencies of $CD4^+$ T-cells among $CD45^+$ leukocytes, and of the indicated conventional T-cell and Treg populations among $CD4^+$ T-cells, in the mesenteric lymph nodes of the Eo-*Cre*^{DTR} mice and their wild-type (Cre$^-$, WT) littermates shown in e-l. Data in p are pooled from two studies. Statistical comparisons were performed by Mann-Whitney (two groups) or Kruskal-Wallis (more than two groups) test followed by Dunn's post-hoc test. * $p<0.05$, ** $p<0.01$, ***$p<0.001$, ****$p<0.0001$. ns, not significant.

Supplementary Figure 2

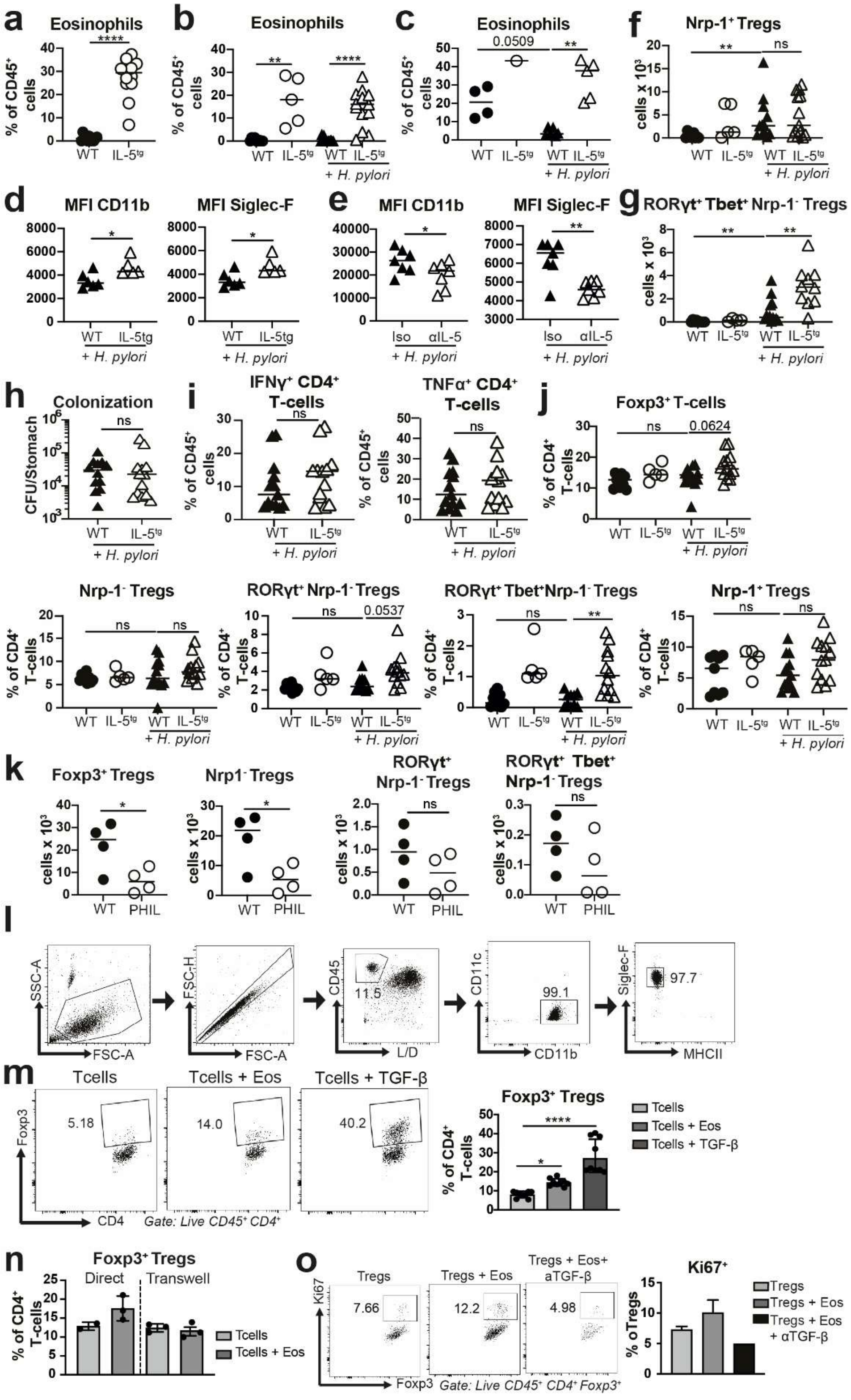

Suppl. Figure 2. IL-5-driven eosinophilia does not affect *H. pylori* immune control despite supporting Treg expansion. a, Frequencies of eosinophils among all blood leukocytes, in IL-5^{tg} mice and their

wildtype littermates. **b,c**, IL-5-transgenic (IL-5^{tg}) mice and their wild-type littermates were infected or not with *H. pylori* strain PMSS1 for six weeks and eosinophil frequencies among all leukocytes were quantified in gastric lamina propria (b) and MLN (c) single cell preparations. **d,e**, Eosinophil activation, as determined by CD11b and Siglec-F staining, of *H. pylori*-infected IL-5^{tg} mice and their wildtype littermates. **f,g,** Absolute counts of gastric lamina propria Nrp-1^+ Foxp3^+ Tregs and of RORγt$^+$ Tbet$^+$ Nrp-1^- Tregs, of the mice shown in main Figure 2a-c. **h**, *H. pylori* colonization as determined by plating and colony counting, of the mice shown in Figure 2a-c. CFU, colony forming units. **i**, Gastric IFN-γ^+ and TNF-α^+ CD4^+ T-cells among all CD45^+ leukocytes, as determined by PMA/ionomycin restimulation and intracellular cytokine staining, of the mice shown in h. **j,** Frequencies of the indicated T-cell and Treg populations among CD4^+ T-cells in the MLNs of the mice shown in h,i. Pooled data from two studies are shown throughout, except in panels c-e which contains data from only one study. **k**, Absolute counts of colonic Foxp3^+ Tregs, Nrp-1^- Tregs, RORγt$^+$ Nrp-1^- Tregs and RORγt$^+$ Tbet$^+$ Nrp-1^- Tregs, of PHIL mice and their wild type littermates. **l,** Representation of the viability and purity of immunomagnetically isolated eosinophils. **m,** Splenic eosinophils immunomagnetically sorted from IL-5^{tg} mice were co-cultured with naïve CD4^+ T-cells from WT mice for three days at 1:1 ratio; Foxp3 expression was quantified by flow cytometry at the study endpoint. Addition of TGF-β to naive T-cells served as positive control. Representative FACS plots are shown alongside a summary plot with pooled data from two independent studies. **n**, Splenic eosinophils immunomagnetically sorted from IL-5^{tg} mice were co-cultured with Foxp3/YFP$^-$ CD4^+ T-cells FACS-sorted from Foxp^{YFP} reporter mice for three days at 1:1 ratio either in direct contact or separated by a trans-well filter; Foxp3/YFP expression as quantified by flow cytometry at the study endpoint. **o**, Splenic eosinophils immunomagnetically sorted from IL-5^{tg} mice were co-cultured with FACS-sorted Foxp3/YFP$^+$ Tregs from Foxp3^{YFP} reporter mice for three days at 1:1 ratio; anti-TGF-β neutralizing antibody was added where indicated. Ki67 expression was quantified by flow cytometry at the study endpoint. Representative FACS plots are shown alongside a summary plot. Data in n and o are from one study each. Statistical comparisons were performed by Mann-Whitney (two groups) or Kruskal-Wallis (more than two groups) test followed by Dunn's post-hoc test. * $p<0.05$, ** $p<0.01$, ***$p<0.001$, ****$p<0.0001$. ns, not significant.

Supplementary Figure 3

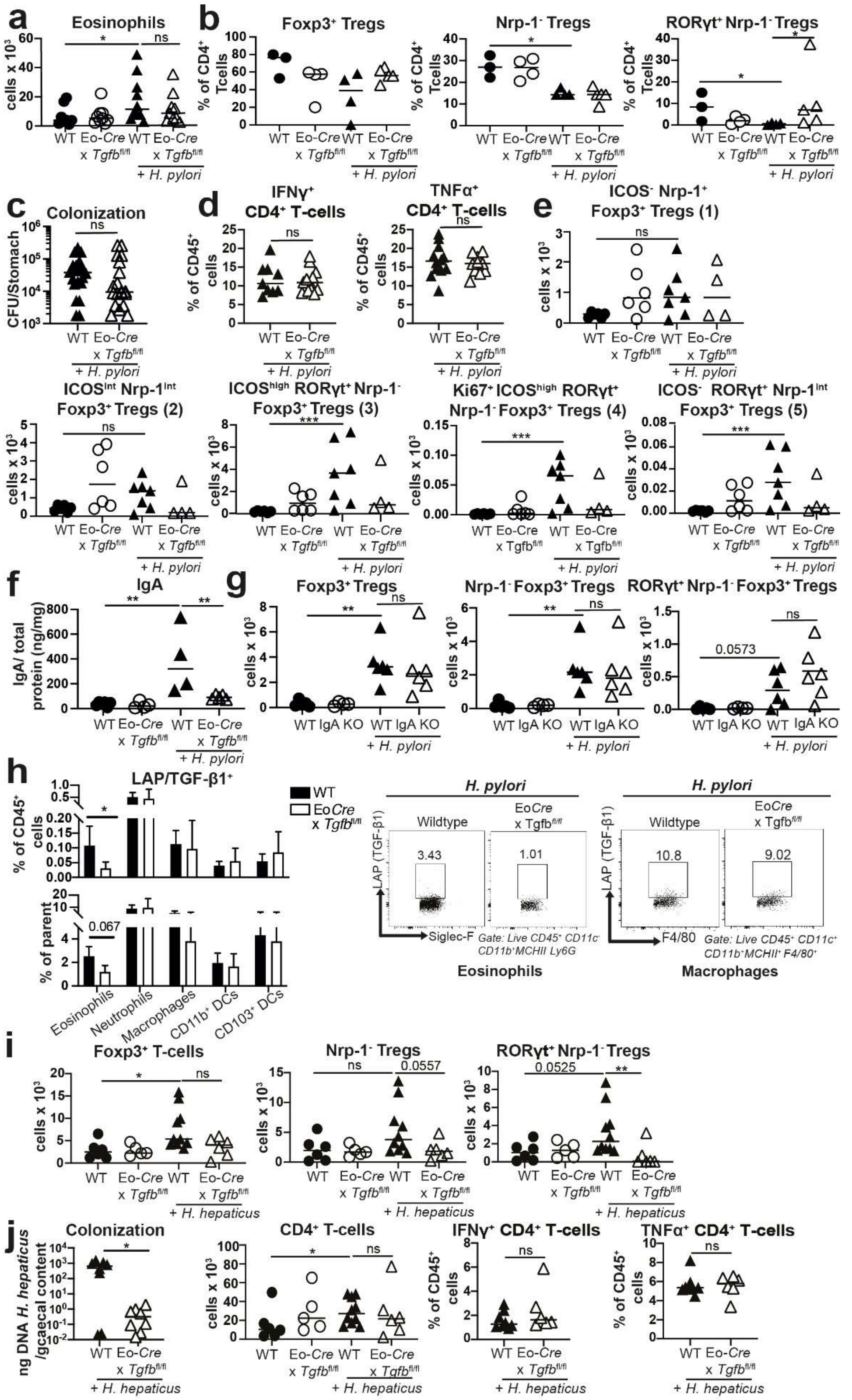

Suppl. Figure 3. The eosinophil-specific loss of TGF-β does not affect *H. pylori* immune control despite compromising Treg expansion. a-d, *Eo*-Cre x *Tgfb*$^{fl/fl}$ mice and their wild-type littermates were

infected with *H. pylori* strain PMSS1 for six weeks. Absolute counts of gastric lamina propria eosinophils are shown in a and frequencies of Foxp3$^+$ Tregs, Nrp-1$^-$ Tregs and RORγt$^+$ Nrp-1$^-$ Tregs in MLNs are shown in b of infected *Eo*-Cre x *Tgfb*$^{fl/fl}$ mice and WT littermates relative to uninfected age-matched controls. *H. pylori* colonization levels are shown in c, and frequencies of gastric IFN-γ^+ and TNF-α^+ CD4$^+$ T-cells among all CD45$^+$ leukocytes, as determined by PMA/ionomycin restimulation and intracellular cytokine staining, are shown in d. Data are pooled from two studies in a and d; data in b are from one representative study of two; data in c are pooled from three studies. **e,** *Eo*-Cre x *Tgfb*$^{fl/fl}$ mice and their wild-type littermates were infected with *H. pylori* for 12 weeks; absolute counts of the five gastric Treg populations identified by spectral flow cytometry based on their Nrp-1, RORγt and ICOS expression are shown for infected mice of both genotypes relative to age-matched uninfected controls. Note that the wild type mice of this cohort are described in more detail in supplementary Figure 1 a-c. **f,** *Eo*-Cre x *Tgfb*$^{fl/fl}$ mice and their wild type littermates were infected with *H. pylori* strain PMSS1 for six weeks. Gastric IgA was quantified by ELISA. **g,** IgA-deficient mice and their wild type littermates were infected with *H. pylori* strain PMSS1 for six weeks. Absolute counts of gastric lamina Foxp3$^+$ Tregs, Nrp-1$^-$ Tregs and RORγt$^+$ Nrp-1$^-$ Tregs are shown. **h,** *Eo*-Cre x *Tgfb*$^{fl/fl}$ mice and their wild type littermates were infected with *H. pylori* strain PMSS1 for six weeks. LAP/TGFβ1 expression by the indicated cell populations was determined by FACS. Representative FACS plots are shown for eosinophils (left panels) and macrophages (right panels) of infected mice of the indicated genotypes. **i-j**, *Eo*-Cre x *Tgfb*$^{fl/fl}$ mice and their wild-type littermates were infected with *H. hepaticus* for six weeks. Absolute counts of colonic lamina propria CD4$^+$ Foxp3$^+$ Tregs, Foxp3$^+$ Nrp-1$^-$ Tregs and RORγt$^+$ Nrp-1$^-$ Tregs are shown in i. Cecal *H. hepaticus* colonization as determined by qPCR is presented alongside the frequencies of colonic CD4$^+$ T-cells, and of IFN-γ^+ and TNF-α^+ CD4$^+$ T-cells among all CD45$^+$ leukocytes, as determined by PMA/ionomycin restimulation and intracellular cytokine staining. Data in i and j are pooled from two independent studies. Statistical comparisons were performed by Mann-Whitney (two groups) or Kruskal-Wallis (more than two groups) test followed by Dunn's post-hoc test. * $p<0.05$, ** $p<0.01$, ***$p<0.001$, ****$p<0.0001$. ns, not significant.

Supplementary Figure 4

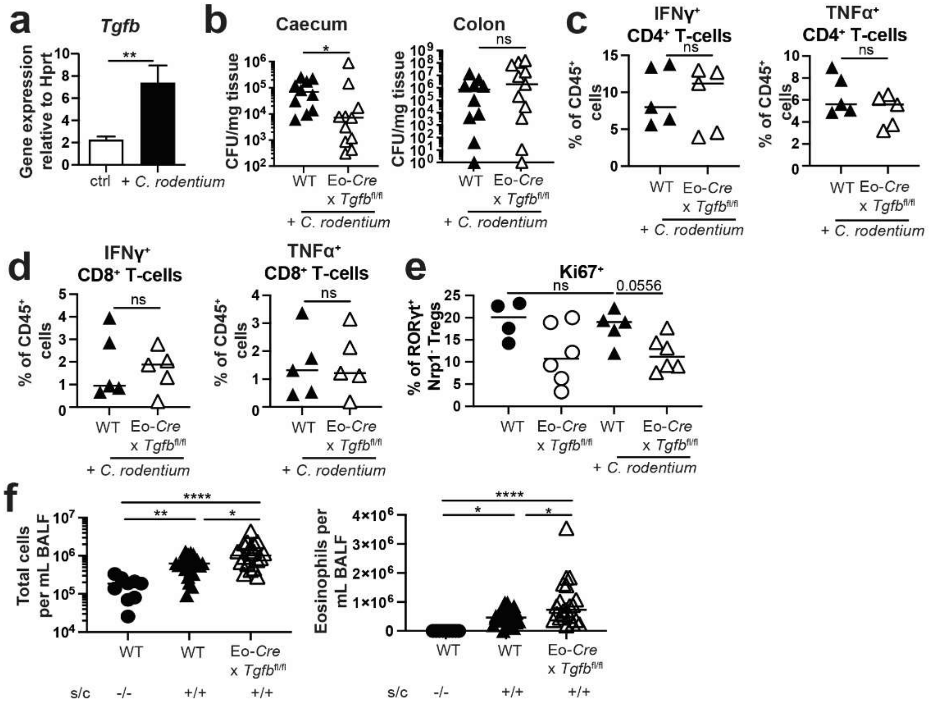

Suppl. Figure 4. The eosinophil-specific loss of TGF-β aggravates type 2 but not type 1 responses. a, Eosinophils sorted from the spleens of IL-5^{tg} mice were exposed to live *C. rodentium* at a multiplicity of infection of 10 for six hours, and subjected to *Tgfb*-specific qRT-PCR. **b-e**, *Eo*-Cre x *Tgfb*$^{fl/fl}$ mice and their wild-type littermates were infected with *C. rodentium* for 12 days. Colonization of the caecum and colon, as determined by plating and colony counting is shown in b, and the frequencies of colonic IFN-γ^+ and TNF-α^+ CD4$^+$ T-cells among all CD45$^+$ leukocytes, and of colonic IFN-γ^+ and TNF-α^+ CD8$^+$ T-cells among all CD45$^+$ leukocytes, as determined by PMA/ionomycin restimulation and intracellular cytokine staining, are shown in c and d. Ki67$^+$ cells among colonic RORγt$^+$ Nrp-1$^-$ Tregs are shown in e. **f**, Mice were sensitized and challenged with house dust mite extract, or mock-treated with PBS only. Bronchoalveolar lavage fluid (BALF) was harvested at the study endpoint, and subjected to differential cell staining using the Microscopy Hemacolor kit. Total cells and eosinophils in 1ml of BALF are shown in f. Data in b are pooled from two studies, data in c-e are from one study, but representative of two, and data in f are pooled from three independent studies. Statistical comparisons were performed by Mann-Whitney (two groups) or Kruskal-Wallis (more than two groups) test followed by Dunn's post-hoc test. * $p<0.05$, ** $p<0.01$, ***$p<0.001$, ****$p<0.0001$. ns, not significant.

Supplemental methods

Culture and quantification of *Helicobacter hepaticus*. *H. hepaticus* ATCC 51449 was a kind gift from Christine Josenhans (Pettenkofer-Institut, Ludwig-Maximilians-University of Munich, Germany). Bacteria were cultured at 37°C under microaerobic conditions, 10% CO_2, 10% H_2, 80% N_2 (CampyPak; Oxoid), on blood agar plates (Columbia agar base II; Oxoid) supplemented with 10% horse blood and antibiotics (10 mg/L vancomycin, 2500 U/L polymyxin B, 5 mg/ L trimethoprim, 4 mg/L amphotericin B). After 2 days, *H. hepaticus* was collected into PBS and centrifuged at 2000 rpm for 5 min. Bacterial pellets were resuspended in 1 ml PBS and suspensions were assessed by light microscopy for contamination, morphology, and motility. Mice were infected intragastrically on two consecutive days with 200ul volume containing approximately $1x10^7$ bacteria. *H. hepaticus* colonization was quantified in cecal contents; genomic DNA was isolated using the DNA Stool kit (QIAGEN) following the manufacturer's instructions. DNA was subjected to quantitative PCR with Taq Man *Hh*-specific primers for the *cdtB* gene (forward: 5'-CCG CAA ATT GCA GCA ATA CTT-3', reverse: 5'-TCG TCC AAA ATG CAC AGG TG-3', probe: 5'-FAM-AAT ATA CGC GCA CAC CTC TCA TCT GAC CAT-MGB-3', ThermoFisher Scientific) [1] and Precision Plus qPCR Master Mix (Primer Design). A standard curve was generated using DNA isolated from plate-grown *H. hepaticus*.

Ovalbumin and house dust mite sensitization and challenge

For ovalbumin (OVA)-induced food allergy, mice were sensitized twice intraperitoneally (i.p.) with 50mg of OVA (Sigma; A5503-5G) emulsified in aluminum hydroxide (Imject alum, 77161; Thermo Scientific) on days 0 and 14, followed by challenge via oral gavage on days 28, 29, 30, and 31 with 60 mg of OVA. Signs of anaphylaxis were scored in a blinded fashion for 30 min after each challenge, with scores indicating the following: 0, no sign of reaction; 1, repetitive scratching and rubbing around the nose/mouth and head and ear canal digging with hind legs; 2, decreased activity with an increased respiratory rate, pilar erecti, and/or puffing around the eyes and/or mouth; 3, labored respiration and

cyanosis around the mouth and tail and/or periods of motionless for more than 1 min or lying prone on stomach; 4, slight or no activity after prodding/whisker stimuli or tremors and convulsion; and 5, death. Cumulative scores over 3 days were calculated by adding all three individual scores per mouse. Mice were sacrificed by CO_2 inhalation after the last challenge, and blood and tissue samples were collected. For splenic antigen-specific Th2 cytokine ELISAs, spleens were pushed through a 40-mm cell strainer and washed with PBS prior to red blood cell lysis. Splenocytes were seeded into 96-well plates in RPMI 1640medium (Gibco 21875-034 plus fetal calf serum [FCS] and penicillin-streptomycin) supplemented with200mg/ml of OVA protein. After 4 days in culture, supernatants were collected and cytokines were quantified by IL-5 (88-7054-88) and IL-13 (88-7137-88) ELISA, according to the manufacturer's instructions(eBioscience). The quantification of OVA-specific IgG1 and IgE was performed by coating high affinity 96-well plates with 100 uL 100 ug/mL OVA in carbonate-bicarbonate coating buffer overnight at 4°C. After washing and blocking, dilutes samples ware incubated for 2 hours, washed again and an HRP-coupled IgE-specific (GeneTex GTX77227) or IgG1-specific (xy) antibody was added for detection. HRP substrate was added and the absorbance was measured on a plate reader. Colonic tissue preparation and Treg staining was prepared as described above and samples were acquired on an LSRII Symphony (BD Biosciences) and analyzed using FlowJo software. For the induction of house dust mite-induced allergic airway inflammation, mice were sensitized on day 0 (1 µg) and challenged on days 8-12 (15 µg) intranasally with house dust mite extract (Greer laboratories, XPB70D3A25 Dermatophagoides pteronyssinus). On day 15, mice were sacrificed, blood was collected and serum prepared. Lungs were lavaged via the trachea with 1 mL of PBS. Broncho-alveolar lavage fluid (BALF) cells were counted using trypan blue dye exclusion. Differential cell counts of macrophages, lymphocytes, neutrophils, and eosinophils were performed on cytocentrifuged preparations stained with the Microscopy Hemacolor-Set (Merck).

Immunofluorescence staining of gastric tissue sections

Paraffin-embedded tissue sections were deparaffinized and rehydrated with graded ethanol dilutions. After antigen retrieval in pressure cooker using sodium citrate buffer (10 mM, pH 6.0), the non-specific binding was prevented by preincubation of the tissue samples with a blocking buffer (containing human immunoglobulins, normal goat serum, and 7.5% BSA in PBS) at room temperature (RT) for 1 h. Indirect immunofluorescence staining was performed by incubating the paraffin sections overnight at 4°C with Primary monoclonal mouse anti-mouse EPX antibody (1:200, clone MM25-82.2; obtained from Lee Laboratories, Mayo Clinic, Scottsdale, AZ, USA), and rabbit monoclonal anti-mouse FoxP3 antibody (1:100; clone D6O8R; Cell Signaling Technology, distributed by BioConcept, Allschwil, Switzerland). Thereafter, secondary Alexa Fluor® 488 - conjugated goat anti-rabbit (1:400), and Alexa Fluor® 545-conjugated goat anti-mouse (1:400) (ThermoFisher Scientific, Reinach, Switzerland) were applied, and tissue samples incubated at RT for 1 h, and stained with Hoechst 33342 solution (5 μM) for additional 10 min. Samples were washed and mounted in Prolong Gold mounting medium and image acquisition was performed using confocal laser scanning microscopy LSM 810 (Carl Zeiss Micro Imaging, Jena, Germany) with a 63x or 40x /1.40 Oil DIC objectives and analyzed with IMARIS software (Bitplane AG, Zurich, Switzerland). For quantitative analysis, $Foxp3^+$ and EPX^+ infiltrating cells were counted in 10 high-power fields (hpf) of highest activity using an automated slide scanner (3DHISTECH slide scanner, Quant Center software, using Cell Count module).

Spectral flow cytometry

Cells were stained using the following directly conjugated antibodies in addition to a live/dead dye. Extracellular: CD45-BUV395, CD4-BUV496, CD44-BUV737, TCRb-PE-Cy5, GITR-APC, Neuropilin-BV421, CD73-APC-C7, CD69-BV605, PD1-BV785, CD39-PerCP-eFluor®710, CD62L-BV570, ICOS-PE, CD27-V450, CD25-BV650; intracellular: FOXP3-PE-Cy7, CTLA4- AF700, Rorgt-PE-

Dazzle 594, Ki67-BV480 (purchased from either Biolegend, Thermofisher, Cell Signaling or BD Bioscience). Samples were acquired on a Cytek Aurora. Umap and FlowSOM metaclustering were performed as described by Brummelman et al.[2]

Eosinophil/T-cell co-cultures

For eosinophil/T-cell cocultures, eosinophils were isolated by positive selection from spleen single-cell suspensions using a PE Siglec-F antibody (Biolegend) and anti-PE microbeads (Miltenyi Biotech). $CD4^+$ $CD25^-$ T cells were isolated by double negative selection from spleen single-cell suspensions using the MagCellect mouse Regulatory T-cell cell isolation kit (R&D Systems). Round-bottom 48-well plates were coated with anti-CD3 antibody (Biolegend) for 4h. Eosinophils and T cells were co-cultured at 1:1 ratio (100,000 eosinophils and 100,000 T cells) in a round-bottom 48-well plate for 72 h in the presence of anti-CD28 agonistic antibody (Biolegend) at a final concentration of 2 µg/ml with 20 ng/ml IL-2 (R&D systems) and 10ng/ml IL-5 (Peprotech). $Foxp3^+$ Tregs were stained for flow cytometry as described in the main methods section, and samples were acquired on an LSRII Symphony (BD Biosciences) and analyzed using FlowJo software.

Supplementary References

1. Ge Z, White DA, Whary MT, Fox JG. Fluorogenic PCR-based quantitative detection of a murine pathogen, Helicobacter hepaticus. *Journal of clinical microbiology* 2001; **39**(7)**:** 2598-2602.

2. Brummelman J, Haftmann C, Nunez NG, Alvisi G, Mazza EMC, Becher B *et al.* Development, application and computational analysis of high-dimensional fluorescent antibody panels for single-cell flow cytometry. *Nature protocols* 2019; **14**(7)**:** 1946-1969.
